# Current Trends in Wireless Mesh Sensor Networks: A Review of Competing Approaches

**DOI:** 10.3390/s130505958

**Published:** 2013-05-10

**Authors:** David Rodenas-Herraiz, Antonio-Javier Garcia-Sanchez, Felipe Garcia-Sanchez, Joan Garcia-Haro

**Affiliations:** Department of Information and Communication Technologies, Technical University of Cartagena (UPCT), Campus Muralla del Mar, Cartagena E-30202, Spain; E-Mails: david.rodenas@upct.es (D.R.-H.); felipe.garcia@upct.es (F.G.-S.); joang.haro@upct.es (J.G.-H.)

**Keywords:** wireless mesh sensor networks, mesh solutions and review, IEEE 802.15.4, IEEE 802.15.5

## Abstract

Finding a complete mesh-based solution for low-rate wireless personal area networks (LR-WPANs) is still an open issue. To cope with this concern, different competing approaches have emerged in the Wireless Mesh Sensor Networks (WMSNs) field in the last few years. They are usually supported by the IEEE 802.15.4 standard, the most commonly adopted LR-WPAN recommendation for point-to-point topologies. In this work, we review the most relevant and up-to-date WMSN solutions that extend the IEEE 802.15.4 standard to multi-hop mesh networks. To conduct this review, we start by identifying the most significant WMSN requirements (*i.e.*, interoperability, robustness, scalability, mobility or energy-efficiency) that reveal the benefits and shortcomings of each proposal. Then, we re-examine thoroughly the group of proposals following different design guidelines which are usually considered by end-users and developers. Among all of the approaches reviewed, we highlight the IEEE 802.15.5 standard, a recent recommendation that, in its LR-WPAN version, fully satisfies the greatest number of WMSN requirements. As a result, IEEE 802.15.5 can be an appropriate solution for a wide-range of applications, unlike the majority of the remaining solutions reviewed, which are usually designed to solve particular problems, for instance in the home, building and industrial sectors. In this sense, a description of IEEE 802.15.5 is also included, paying special attention to its efficient energy-saving mechanisms. Finally, possible improvements of this recommendation are pointed out in order to offer hints for future research.

## 1. Introduction

The IEEE 802.15.4 specification [1] is the most widely adopted point-to-point communication standard for low-rate wireless personal area networks (LR-WPANs). Its success lies in the design of a very straightforward protocol stack comprising the physical (PHY) and medium access control (MAC) layers, both developed for low-cost, low-power and resource-constrained wireless devices. The goal of this standard is to provide an appropriate solution for a few devices/nodes deployed in a small-sized area. Devices acquire data of interest (monitoring one or several physical parameters) and transmit them to their destination (sink) periodically or in event-driven fashion. Under these conditions, this standard exhibits two main limitations: (1) short-range communications with a small bandwidth available (maximum bitrate of 250 Kbps at 2.4 GHz) and (2) lack of multi-hop communications and mesh capability. In particular, mesh capability is understood here as the suitable provision/promotion of a set of features such as scalability, reliability, robustness, security, connectivity or coverage area in wireless mesh topologies, but always having into consideration the principle of energy efficiency [2]. These drawbacks reveal how IEEE 802.15.4 is unable to satisfy current demands for wireless sensor communications aimed at reaching a right solution for long-term monitoring over large areas. Nevertheless, the main commercial Wireless Sensor Network (WSN) manufacturers have employed this recommendation (or some of its functionalities) to develop their own standards and proposals. Those manufacturers that have developed IEEE 802.15.4-based solutions including mesh capability and multi-hop communications will obtain a twofold benefit: on the one hand, a notable improvement with respect to the traditional already deployed WSNs, and, on the other hand, the ability to plan innovative LR-WPAN applications/services on a multiplicity of different market segments, comprising automation and control (home and industrial), environmental surveillance, precision agriculture, traffic monitoring, and health services, among others.

All these issues are reflected, for instance, in the trend shown by the industrial automation market in the last few years. According to a recent report published by On World [3], up to 75% of 216 surveyed industrial automation professionals/end-users (many of them, well-known companies/manufacturers) have already installed or plan to deploy solutions based on WSNs to cope with their needs. In particular, 57% of the end-users are currently using or pilot testing WSN systems. Seventy-five percent of the current WSN end-users indicate they are considering employing the IEEE 802.15.4 standard, comprising mesh capability, for at least some of their deployments, while the 20% of the surveyed professionals reported that they intend to exclusively use mesh solutions for LR-WPANs. Furthermore, the On World study points out that advances on wireless mesh sensor technology over the next few years will assure a strong expansion of this technology in the consumer market. In this sense, the On World study foresees that, in 2016, around 39% of the wireless devices will be used for new applications and services that are uniquely enabled by LR-WPAN mesh networks. Therefore, we observe a clear migration from conventional LR-WPAN networks composed of only a few nodes to large-scale deployments where all devices communicate with each other by means of a standard mesh solution.

Thereby, in order to provide traditional LR-WPANs [4] with mesh capability, in the last few years the scientific community has mainly concentrated its efforts on the design and development of upper layers on top of the IEEE 802.15.4 protocol stack. The goal is to generate multi-hop large-scale networks, encompassed into the Wireless Mesh Sensor Networks (WMSNs) technology, in which any source device is able to dispatch data to a destination (or several ones) out of its coverage range, by selecting the best path of intermediate nodes between both end nodes. In addition, as a well-known benefit, a WMSN balances the network traffic load with the aim of avoiding, among other concerns, bottleneck nodes or information losses when an intermediate node dies. This fact reveals that a WMSN achieves much better network performance than the traditional WSN-based implementations restricted, often, by the topology (usually, star or tree topologies). However, successfully running LR-WPAN applications that support mesh capability implies a notable waste of energy on communication issues (*i.e.*, generation of the mesh network or efficient data transmission/reception among intermediate nodes belonging to a path). This translates in a reduction of the devices' lifetime (time interval since a device is activated for first time until it depletes its batteries), which complicates long-term solutions. To overcome this, devices belonging to a WMSN must be efficiently self-organized in a mesh topology (regardless of the number of devices and transparently to users) and must operate coordinately to carry out efficient communications. This can be reached through the careful implementation of communication software modules into the resource-constrained devices, considering: (1) their energy-constrained premises (devices generally equipped with a short number of chemical batteries, and placed at isolated locations where the use of external power supply is often unfeasible) and (2) their well-known hardware restrictions of memory and processing capabilities. Thereby, straightforward solutions must be designed and developed to offer the best performance for mesh topologies in terms of scalability, interoperability, mobility, self-organization, reliability, robustness, security or connectivity, among others; without jeopardizing the primary objective of low power consumption [5]. In this sense, an appropriate scheduled operation (comprising active and inactive periods –ON/OFF–) would guarantee an extension of the devices' as well as the network's lifetime, being the network's lifetime the time interval since the network starts its operation until one or several nodes deplete their batteries, making the network operation unfeasible. Currently, this type of solutions has not been completely fulfilled by any WMSN proposal or standard, what makes it an open challenge for the scientific and manufacturer communities.

In this context our paper has three main contributions. First, we review the current WMSN standards and proposals that facilitate the development of mesh topologies for LR-WPANs. To this end, we have identified the most relevant WMSN requirements with the aim of learning about the pros and cons of each proposal. The goal is to discern what issues prevent the complete penetration of WMSNs in the market. Regarding these proposals, several organizations and industrial alliances, such as IEEE, Zigbee® Alliance, IP500® Alliance, the Internet Engineering Task Force (IETF), the Highway Addressable Remote Transducer (HART) Communication Foundation (HCF), or the International Society of Automation (ISA) have promoted their own commercial standards but, unfortunately, most of them lack some significant features (e.g., energy saving mechanisms or interoperability with other wireless technologies) or are simply not validated by a thorough and complete study or evaluation, which means that its true performance is in fact unknown. Despite the existence of available studies comparing some of these proposals [4,6–8], no research work jointly deals with all of them or focuses particularly on the WMSN field. Therefore, to the best of our knowledge, this is the first study that addresses these concerns, which means a significant step forward for this technological area. Additionally to this first study and as our second contribution, we have deeply examined each of the solutions according to different design guidelines. We have selected these guidelines since they are usually taken into account by the scientific/developer community [2,9,10] and different well-known end-users [3] in accordance with the WMSN requirements with the aim of selecting the best solution for their application. As a result of these works, none of these proposals satisfactorily solve most of the LR-WPAN requirements in mesh topologies. Under these circumstances, IEEE recently published the IEEE 802.15.5 standard, a recommendation that extends the IEEE 802.15.4 functionality to the WMSNs arena. This standard offers the user multiple valuable services, highlighting among them two efficient energy saving mechanisms. In comparison with the rest of proposals, IEEE 802.15.5 addresses the largest number of WMSN requirements and design guidelines, what makes it an appropriate candidate for a wide-range of WMSN applications. This is a clear advantage over most of the approaches studied in this paper, which are usually designed to solve some particular problem in the home, building and industrial sectors. Therefore, as a third contribution of this paper, we discuss the fundamental features and operation details of the IEEE 802.15.5 standard focusing on its advantages in comparison with the rest of commercial WMSN proposals. However, not all are benefits in this standard. In particular, some features such as the end-to-end reliability or security are not specified, and researchers and developers currently lack an available open-access implementation of this standard as well. These are the reasons why we also provide some hints aimed at laying the basis for future improvements of this standard.

The rest of the paper is organized as follows: the main requirements of WMSNs are identified and outlined in Section 2. Then, in Section 3, we review the most relevant contending proposals in the WMSN area. Section 4 introduces the IEEE 802.15.5 standard and its two energy saving mechanisms. Section 5 discusses the proposals selected as a function of different design guidelines. Next, in Section 6, we highlight the benefits related to the IEEE 802.15.5 standard and point out its open research issues. Finally, Section 7 concludes the paper.

## 2. Wireless Mesh Sensor Network Requirements

Providing conventional LR-WPANs with mesh capability is crucial for the commercial expansion of this technology, as well as for generating new applications or niche markets (*i.e.*, protection firefighter operations, multimedia services, situational awareness and precision asset location, or security and environmental monitoring, among others). To achieve this goal, the WMSN design phase must consider several significant *requirements* influencing the performance of WMSNs. They are summarized as follows:

(1) *Energy-management policy* and *energy-efficient design*: This requirement is a critical issue in the design of WMSNs. Devices are usually fed by AA/AAA chemical batteries, which sharply restricts the life of network nodes in the case of continuous operation. Furthermore, in many occasions, the use of external power supply sources (e.g., solar or wind) is too costly or, simply, unfeasible for the type of application or emplacement. These concerns are even more decisive in large-scale mesh networks because the number of hops between source and destination (multi-hop) may be high, increasing the energy-demands associated to data transmissions/retransmissions, and therefore shortening network lifetime. Energy-management mechanisms alleviate these concerns as they reduce the power consumption of nodes by means of, for instance, the temporary disconnection of specific hardware components such as the CPU or the radio transceiver. Nevertheless, the rest of node's activities (such as routing procedures, addressing scheme, or security) must also be carefully designed to avoid nodes overload, what also increases the energy demands.
(2) *Link reliability*: One of the most important factors when designing WMSNs is ensuring the permanent link connectivity between source-destination pairs during the entire operation of the application. In contrast to other topologies (*i.e.*, the tree topology) a mesh network guarantees link reliability through the possibility of multiple paths connecting sources and destinations. This, in turn, enables alternative routes in the case of, for instance, a dead intermediate node. Furthermore, this requirement can be improved by the increase in the number of devices in a delimited sensing area, what implies greater path redundancy. This means an improvement in the connectivity for all nodes belonging to the area under consideration because they have a greater number of neighbors in coverage area.
(3) *Robustness*: A WMSN must furnish fault tolerance and self-healing features in order to cope with concerns, such as topology changes [e.g., nodes (dis)appearing], aggressive dynamic environmental conditions or radio interferences. In particular, interference may be caused by different phenomena: network devices sharing the same broadcast medium or external signals coming from other wireless technologies operating in the same band, such as Wi-Fi. Therefore, this requirement may be clearly improved by exploiting the path redundancy of the network and by using efficient mechanisms that mitigate or eliminate such effects.
(4) *Scalability support*: This requirement pursues the non-degradation of the network performance as the number of devices grows. The goal of WMSNs consists of extending the coverage range of traditional WSNs through networks formed by hundreds or even thousands of battery-powered devices. Thereby, scalability becomes a decisive requirement for large-scale monitoring applications in order to guarantee robustness, link reliability and an efficient consumption of energy.
(5) *Interoperability*: A searched feature of WMSN protocols is that they guarantee the compatibility and inter-connection among heterogeneous networks including other wired and wireless technologies. To this end, interoperability must be provided without increasing the complexity of the software and hardware modules. A WMSN solution based on the IEEE 802.15.4 protocol stack would satisfy, *a priori*, the compatibility with other solutions which fully employ this same standard, and the inter-connection with multiple wired/wireless technologies by means of commercial gateways available.
(6) *Self-organization*: WMSNs should be easily configured and maintained requiring little or no human intervention. This requirement is one of the most attractive aspects of the WMSNs for the end-user because it demands a very low effort in network maintenance tasks. However, self-organization is also one of the major challenges for developers. The reason is that nodes must implement enough intelligence and autonomy (by means of efficient software/hardware modules) to auto-organize themselves in a mesh topology. Nevertheless, this fact entails a significant trade-off with the energy efficiency.
(7) *End-to-end reliability*: This requirement guarantees the correct information delivery at destination. As discussed before, link reliability can be improved by increasing the number of devices within the sensing area as well as the path redundancy. This issue allows us to reduce the ratio of messages lost in WMSNs (caused, for instance, by path loss fading in the wireless propagation medium). However, some applications demand transport layer services to assure the information delivery (e.g., precision asset location or personal health monitoring) or, at least to notify any important event occurred in the network at destination (e.g., detection of intruders in a restricted zone). Therefore, techniques such as the support for data prioritization can be applied to enhance the end-to-end reliability of the network.
(8) *Security*: Security is a key issue in WMSNs because wireless communications can be easily eavesdropped, altered (replacement, deletion and/or injection of data) and disrupted by malicious attackers from inside/outside the network in absence of security mechanisms. For instance, a dishonest user can keep sending a large volume of queries to the network to prevent authorized users from accessing the sensor data. Thereby, the main security objectives consist of guaranteeing the *confidentiality* and *integrity* of data; the *authentication* to verify the identity of nodes; and the *availability* to ensure the survivability of the application facing attacks that disrupt the right operation of the network.
(9) *Mobility support*: The majority of WSN and WMSN applications are planned for static nodes, where they usually remain in the same initial location. However, some applications require mobility support of sinks and/or sources. For instance, in large-scale networks, sinks can be displaced along different strategic points to ensure the delivery of data from the sources as well as to improve different metrics such as the transmission delay.

## 3. Recent Advances on IEEE 802.15.4-Based WMSN Approaches

**Figure 1.**
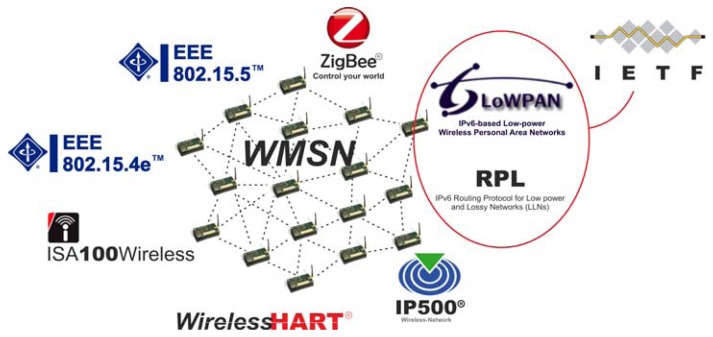
Most relevant standards and proposals which provide LR-WPANs with mesh capability.

This section summarizes the current state of the art focusing on the most relevant approaches providing conventional LR-WPAN with mesh capability. Special attention is paid to the advantages and shortcomings of the different proposals reviewed. Among them, we should highlight the most extended commercial WMSN solutions which are illustrated in Figure 1, namely: Zigbee Pro [11], the IETF group with its solution [12], which encompasses the IPv6 over Low power Wireless Personal Area Networks (6LoWPAN) [13] and the IPv6 Routing Protocol for Low Power and Lossy Networks (RPL) [14], IP500 [15], WirelessHART [16], and ISA SP100.11a [17]. In addition, the recent IEEE 802.15.4e MAC standard [18] is also discussed, because despite not being strictly a WMSN solution, it can be interpreted as the underlying support for future mesh proposals. Finally, although out of the scope of the above mentioned solutions, the IEEE 802.15.5 standard [19] is a novel WMSN standard that demands attention as well, therefore it is further described in the next Section.

**Figure 2.**
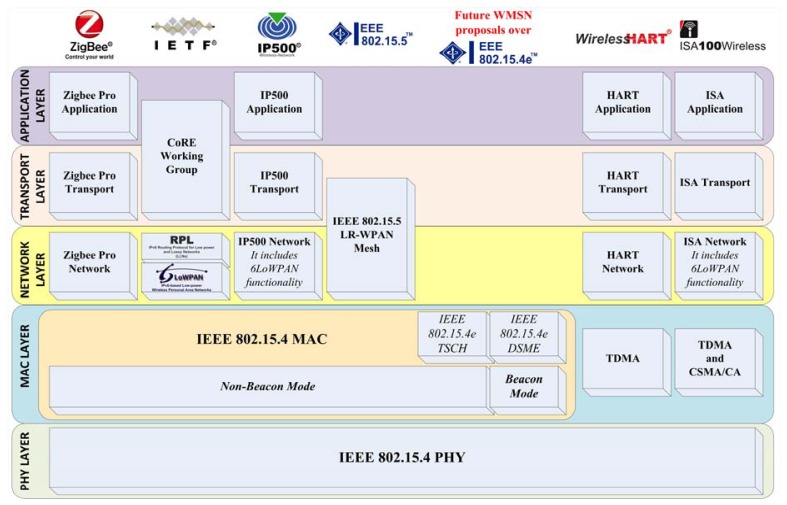
LR-WPAN architecture for the current wireless sensor approaches enabling mesh deployments.

Figure 2 shows a general snapshot of the protocol stack referred to the WMSN solutions under consideration. Observing this Figure, all these proposals share a common starting point: they fully or partially implement the recommendations of the IEEE 802.15.4 standard for their respective PHY and MAC layers. Studying deeply each one of these proposals, they can be classified into two main categories: (i) those that only use rules related with the IEEE 802.15.4 PHY layer, and (ii) proposals that employ both, the MAC and PHY layers of this standard.

Regarding the first category, WirelessHART and ISA SP100.11a are two specifications which integrate in their implementations some foundations of the IEEE.802.15.4 PHY layer. The second category comprises proposals that share the same frame format (thus contributing to their interoperability) but differ in the use or not of scheduling mechanisms at the IEEE 802.15.4 MAC layer. Then, the second category is further subdivided into two groups as follows: *non-beacon mode* and *beacon mode* solutions. Following with this sub-classification, the first group, the IEEE 802.15.4 MAC non-beacon mode solutions, is characterized by transmitting messages according to the unslotted Carrier-Sense Multiple Access-Collision Avoidance (CSMA-CA) algorithm [20]. It presents a higher flexibility for the transmission operations of the mesh network but at the expense of deteriorating other network performance figures, such as the message delay in conditions of high network traffic. As illustrated in Figure 2, WMSN solutions such as Zigbee Pro, 6LoWPAN/RPL, IP500 or IEEE 802.15.5 fit in the non-beacon mode at MAC layer category. Finally, in this group, we have also included a mechanism belonging to the IEEE 802.15.4e standard called Time Slotted Channel Hopping (TSCH) [18], which provides the basis for future WMSN implementations in non-beacon mode.

On the other hand, the IEEE 802.15.4 beacon mode category transmits information within strict temporal intervals. In the beacon mode, a LR-WPAN is created and managed by a special device called PAN Coordinator. The PAN Coordinator has a double functionality: (i) it usually acts as destination/sink of the information and (ii) it coordinates the access to the physical medium of the remaining network nodes. The PAN Coordinator periodically transmits *beacon frames* (specific messages including control information, *i.e.*, the operation channel, address identifier, *etc.*) informing the network nodes about the beginning of the so-called *superframe*, a time interval divided into a slotted active period –ON– and an inactive period –OFF– (this ON/OFF operation is also known as duty-cycle). Thus, nodes listen to the medium for the reception of these *beacon frames*. When it occurs, network nodes can transmit data (during the active period) as follows: (i) by using the slotted CSMA-CA algorithm [21] and/or (ii) by reserving dedicated slots in order to guarantee the data delivery at destination fulfilling strict temporal requirements, thus increasing the network reliability. When the active period ends, nodes switch to a low-power state (the sleep state) to save energy. However, the main disadvantage lies in the difficulty to extend the traditional one-hop IEEE 802.15.4 topologies to multi-hop mesh. In this sense, if we assumed a mesh network running under beacon mode, we would require intermediate devices to retransmit the sensed data when sources (usually, end-devices) and sinks are mutually out of coverage. This requires that intermediate nodes perform a similar functionality to that of the PAN Coordinator, that is, they must also dispatch beacon frames (hence acting as *coordinator devices*) in order to coordinate the access to the medium of other intermediate nodes and/or end-devices in their coverage range. In this case, the lack of an appropriate coordination mechanism taking into account the PAN and the *coordinator* nodes may provoke collisions of beacon frames (and also of data messages) and, therefore, message retransmissions or even losses, deteriorating the network performance [22]. Currently, to the best of the authors' knowledge, there is no WMSN implementation using the beacon mode of the IEEE 802.15.4 standard. However, we should mention the IEEE 802.15.4e standard, a recent MAC solution that incorporates a mechanism denoted as *Deterministic and Synchronous Multi-channel Extension* (DSME) [18], which solves the coordination of the nodes in a mesh network by means of the beacon mode scheme. The goal of this mechanism is to set the premises for designing future WMSN proposals over IEEE 802.15.4 PHY/MAC in beacon mode.

### 3.1. WMSN Solutions Developed on Top of the IEEE 802.15.4 PHY Layer: WirelessHART**^®^** and ISA™ SP100.11a

WirelessHART [16] and ISA SP100.11a [6,17] are two WMSN standards which have been released by the HART Communication Foundation (HCF) and International Society of Automation (ISA) groups, respectively, and which use only some functions of the IEEE 802.15.4 PHY layer. Both solutions are intended for the industrial sector, in particular, for control and automation processes. In the case of LR-WPAN, WirelessHART protocol stack [6,16,23,24] implements some features of the IEEE 802.15.4 PHY layer, providing its own implementation for the rest of this and other upper layers (link, network, transport and application). WirelessHART employs a modulation based on a combination of Direct-Sequence Spread Spectrum –DSSS–, and Frequency-Hopping Spread Spectrum –FHSS– at the PHY layer in order to assure efficient data transmission and to be more robust against interferences. Furthermore, a Time-Division Multiple Access (TDMA) scheme [25] strictly controls the access of the messages generated by neighbors in coverage to the medium, offering deterministic latencies and minimizing the number of collisions. This TDMA mechanism allows WirelessHART devices to switch, in non-active periods, to the sleep mode (OFF) in order to extend the node's lifetime. Regarding the upper layers of WirelessHART, they include the proprietary HART specification (last version named HART 7). In particular, as regards the network layer, WirelessHART uses a central node, denoted as Network Manager (NM), intended to create the network and to establish and maintain all the source-destination paths. To this end, the Network Manager needs to discover the current network topology. Additionally, continuous information is collected by this node to detect any change occurred in the different network paths. This type of design through a central node implies a clear increase in the traffic overhead (additional traffic, mainly control messages and retransmissions, generated to send data messages in multi-hop networks) in the network and, consequently, in the energy consumption, particularly on those nodes close to the Network Manager. Furthermore, the NM centralizes the decision making in cases such as when communication errors occur because of interferences, high traffic load or topology changes (e.g., node failures), eliminating this functionality for the remaining network nodes. A weakness of WirelessHART refers to its lack of interoperability with other WMSN based on IEEE 802.15.4, mainly because the MAC layer designed by WirelessHART is incompatible with the IEEE 802.15.4 MAC layer. Finally, it should be noted that WirelessHART components require more hardware complexity to support the full functionality of the standard, which means an extra cost.

On the other hand, ISA SP100.11a [6,17,26–28] offers the industrial sector efficient communications in an interfering environment by means of the implementation of specific software modules that minimize the number of collisions among messages and provide security and deterministic latencies on the data transmission. As WirelessHART, it also uses DSSS/FHSS at PHY layer, and TDMA at MAC layer. Unlike WirelessHART, ISA SP100.11a includes the CSMA-CA protocol at MAC level to further enhance the protection against collisions in the network. Additionally, routing paths are also configured by a Network Manager, but, in comparison with WirelessHART, ISA increases the number of redundant paths between a same source-destination pair. As it can be seen, WirelessHART and ISA SP100.11a share a significant number of features at PHY and MAC levels [27], what has contributed to the recent establishment of a work group, denoted as ISA 100.12 [6], aimed at integrating WirelessHART and ISA SP100.11a standards in a single recommendation. ISA SP100.11a supports both 16-bit-based and 64-bit-based addressing schemes. Furthermore, ISA headers can exploit a 128-bit addressing scheme to enable IP connectivity inside the mesh network. This is possible through the addition of 6LoWPAN standard (described in the next subsection) within the ISA network layer. The main drawbacks of this standard, as well as in the case of WirelessHART, are, on the one hand, the high implementation complexity of the entire protocol stack, what requires more advanced and costly devices and, on the other hand, the lack of interoperability with other WMSNs based on IEEE 802.15.4 standard. From the developers point of view, this fact might be incompatible with the deployment of low-rate and low-cost WPANs, therefore restricting its use and future penetration in the market. Finally, WirelessHART and ISA SP100.11a are proprietary standards, what means that both specifications and implementations are only accessible by payment.

### 3.2. WMSN Solutions Developed on Top of the IEEE 802.15.4 PHY and MAC (Non-beacon Mode) Layers

#### 3.2.1. Zigbee^®^ Pro

Regarding the non-beacon mode category at MAC layer, Zigbee^®^ Alliance is one of the greatest players in the WSN market due to its Zigbee standard, released in 2004 (later revised in 2006), and to Zigbee Pro (2007), both mainly addressed to the industrial market. These specifications [11] coincide in that both implement the upper layers over the protocol stack of the IEEE 802.15.4-2003 recommendation. However, the major difference between both standards is in the topology supported: while Zigbee implements cluster-tree scenarios [29], Zigbee Pro sets the rules to generate and operate mesh networks. Then, in line with our work, we focus on discussing the Zigbee Pro [28,30,31], and, in particular, how it operates in order to deploy WMSNs. Under ZigBee Pro, devices are identified by two different types of addresses: a MAC address (based on 64-bit IEEE), and a logical address (based on 16-bit IEEE). Each network device exclusively has a MAC address which is employed for the formation of the mesh topology. In this same phase, each node is assigned a specific logical address in order to later perform the communication tasks. Note that by making use of a short address scheme (16 bits), devices demand less memory and computing processing resources to create and manage the routing tables. However, one of the main disadvantages of Zigbee Pro lies in its address assignment mechanism. It follows a random procedure in which each device individually obtains a logical address from an available set. In particular, this set is the same for all network nodes, what may provoke that two or more devices get the same logical address. This fact generates conflicts in the operation of the network, concern known as *duplicate addresses*. In order to overcome it, Zigbee Pro devices execute an address resolution mechanism based on the employment of MAC addresses instead of the logical ones in order to later accomplish communications. However, this solution negatively impacts the nodes because of the significant increase in memory utilization. In any case, the major concern of Zigbee Pro (and also Zigbee) standard is related with its Ad-hoc On-demand Distance Vector (AODV) [32] routing protocol. Due to its reactive nature, *AODV does not guarantee network scalability* and it is unable to distribute the traffic load efficiently. This may generate bottlenecks which, among other problems, lead to a greater waste of energy in those devices involved in the bottleneck than in the remaining network nodes. This circumstance is even worse because *Zigbee Pro lacks energy-saving mechanisms*, deteriorating the lifetime of the Zigbee Pro nodes and, in general, of the entire mesh network. As regards the public accessibility, Zigbee and Zigbee Pro are open-access standards [11], although contributions addressed to developing new functionalities are only available by fee payment. Finally, despite the fact that all these drawbacks have had an important influence on the reduction of the number of Zigbee's users in the last years, this recommendation has currently a dominant position in the WSN and WMSN markets.

#### 3.2.2. IETF: 6LoWPAN and ROLL

The Internet Engineering Task Force (IETF) and, in particular, its three work groups: 6LoWPAN [13], Routing Over Low-power and Lossy networks (ROLL) [14] and Constrained RESTful Environments (CoRE) [33] play an important role in the contribution to finding a suitable WMSN solution. The goal pursued is the adaptation of the IPv6 Internet protocol onto the IEEE 802.15.4 standard MAC (in its non-beacon mode) and PHY layers [12]. The work in [12] shows how CoRE, 6LoWPAN and ROLL groups work on a complete solution to offer Internet connection to WMSN devices mainly at home, building and industrial scenarios. In particular, CoRE defines different aspects of the IP-based application and transport layers, delegating the addressing, header compression and fragmentation tasks to the 6LoWPAN association and the routing issues to the ROLL group. Likewise, 6LoWPAN and ROLL develop an efficient solution to implement the network layer capabilities into the WPAN devices. The 6LoWPAN deals with this issue integrating IPv6 datagrams within the frame format of the IEEE 802.15.4 standard. To this aim, 6LoWPAN employs header compression and fragmentation techniques to reduce the 1,280 bytes of the IPv6 maximum transmission unit (MTU) to the available payload of an IEEE 802.15.4 frame (maximum 118 bytes at PHY layer) [34]. However, implementing these techniques implies in fact a high computational cost (CPU, memory and energy consumption) for the resource-constrained devices, which is observed as a weakness of 6LoWPAN [35]. Regarding the addressing scheme, each network node is identified by a unique 128-bit IEEE logical address (IPv6 address). To generate this IPv6 address, 6LoWPAN offers the developer two procedures: (i) the inclusion of an IPv6 prefix to the 64-bit IEEE MAC address of each node, thus extending the header size and (ii) the addition of a Dynamic Host Configuration Protocol version 6 (DHCPv6) server in the network, which in turn involves an extra cost. In both cases, the use of IPv6 addresses implies a clear disadvantage in terms of increasing memory and processing resources when mapping (generating and handling the routing tables) every neighbor node to its respective IPv6 address.

On the other hand, ROLL is in charge of developing routing tasks and solving the multi-hop communications among network devices through the RPL routing protocol [12,36]. In particular, RPL enables large-scale deployments, but unfortunately, communications are based on tree-based routing. This means that every node only communicates with its parent and/or children nodes, what, on the one hand, *reduces the link reliability and robustness of the network* when, for instance, a parent is dead; and, on the other hand, it is prone to bottlenecks (no alternate paths different to tree routes). Another RPL disadvantage is related to the need to be further tested and evaluated on large-scale networks. This is an issue about which, as far as we know, no research work has been published yet. Finally, both 6LoWPAN and ROLL specifications *lack energy saving mechanisms*, which is a critical weak aspect for long-term WMSN deployments. In order to overcome it, the authors of works in [12,34] propose an asynchronous duty-cycle scheme that periodically listens to the physical medium to determine if there is any ongoing transmission (technique known as *low-power listening*). In particular, nodes are woken up at periodic intervals to sense the medium; they activate the radio to hear for any communication activity. This activity is started by a sender dispatching a message called *preamble*, which contains control information about, for example, the type of transmission (unicast or multicast), addressing information or the specific receiving node/-s of the data. On the other hand, all nodes placed in the sender's coverage area must be active to receive the *preamble*. The information in the *preamble* reveals the destination/-s node/-s that must remain in active mode to receive data, while the rest of nodes switch to the sleep state to save energy. The main drawback of this approach is the extra-energy wasted by senders to transmit the *preambles* and by receivers which must activate their radio transceiver a time interval defined by the user to receive it. To conclude, we refer hereafter to the IETF solution composed of 6LoWPAN and RPL as *6LoWPAN/RPL*.

#### 3.2.3. IP500^®^ Alliance

Recently, another IP-based mesh solution was provided by the IP500^®^ Alliance [15], an industrial association driven by important Original Equipment Manufacturers (OEMs) such as DORMA, Gunnebo, JOB Detectomat and Honeywell, among others. The IP500^®^ Alliance is focused on diverse market segments such as home, industrial or building automation. Its goal is to provide a solution that guarantees interoperability and low-power consumption to end-users (*i.e.*, OEMs or system manufacturers) by means of a full protocol stack based on well-accepted standards, regulations and industry protocols for WMSNs. In particular, the IP500 specification employs the MAC and PHY layers (only in the 433 MHz, 868 MHz and 915 MHz bands) of the IEEE 802.15.4-2006 standard and the operation of 6LoWPAN. Furthermore, as an added-value function, this approach offers support to the application layer for building automation and control network solutions through implementations as the BACnet™ protocol [37]. IP500 implements a 16-bit IEEE addressing scheme to support communications between WMSN nodes. Additionally, IP500 also offers the network layer the complete functionality of 6LoWPAN for providing direct connectivity among WMSN nodes and IP-based devices. In contrast, 6LoWPAN/RPL always employs IPv6 addresses regardless of the type of device. As a result, in peer-to-peer WMSN communications, IP500 routing tables and message headers are lighter than under 6LoWPAN/RPL, what guarantee**s** most efficient communications and generate**s** less traffic load on the network. Nevertheless, the IP500 proposal requires nodes with enough memory and processing capabilities to include all the 6LoWPAN functionality (and other implementations as BACnet™) into resource-constrained devices. On the other hand, IP500^®^ Alliance *indicates that only end-devices can implement an energy-saving mechanism based on duty-cycling, while the rest of nodes remain always in active mode*. This makes the IP500 approach unfeasible as a valid solution for applications with energy-constraints (*i.e.*, only battery-powered devices). However the IP500^®^ Alliance is still in its early stages of design and development, therefore an open-access standard allowing a more detailed study of this proposal is not available yet.

#### 3.2.4. IEEE 802.15.4e: The TSCH Protocol

As a particular proposal in this group of solutions, we should also mention the TSCH protocol, which is founded on the design of an *ad-hoc* TDMA-based schedule over the IEEE 802.15.4 non-beacon mode. TSCH is included in the IEEE 802.15.4e specification; a MAC mechanism aimed at amending the last revision of IEEE 802.15.4 standard (released in 2011 [38] to include in a same document the IEEE 802.15.4-2006 together with the PHY amendments published in 2007 and 2009) to offer support to the industrial market segment. IEEE 802.15.4e is not a complete WMSN solution because it does not have upper layers on-top of the MAC level which would provide, for instance, mesh routing capabilities. Nevertheless, this standard furnishes mechanisms such as TSCH and DSME (described in the next subsection), in order to offer support for future WMSNs.

One of the main advantages of TSCH is the use of the technique denoted as channel hopping, which consists of switching dynamically among the 16 available channels of IEEE 802.15.4 PHY layer at running time [18]. The goal of this technique is to mitigate interferences of devices belonging to the WMSN transmitting in the same coverage area and the effect of path loss fading, what in turn facilitates simultaneous transmissions in the vicinity. This is advantageous in relation to the traditional IEEE 802.15.4 standard because it only allows us to select different frequency channels at the network initialization phase in order to avoid interferences with other existing WPANs. Furthermore, this frequency-diversity scheme along with its TDMA-based technique may be very useful for the WirelessHART and ISA SP100.11a proposals, since the scheduling and modulation they implement are fully compatible with TSCH. Therefore, WirelessHART and ISA SP100.11a proposals could improve their interoperability in the industrial field by employing the TSCH mechanism.

In TSCH, a set of nodes in coverage range are synchronized [39] by means of a periodic time-slotted structure known as *slotframe*. Each slot belonging to the *slotframe* is long enough to allow the communications between two nodes, in particular, the transmission of one data message and its corresponding acknowledgement. Going into more detail, the operation of TSCH is based on the fact that devices reserve one slot for receiving messages within the *slotframe* structure. It implies that, before transmitting a message, a sender must learn about the following two issues: (1) the beginning of the receiver's reception slot; and (2) the channel employed by the receiver [18]. In particular, a different channel known by sender and receiver is selected on every new slot. To this end, both nodes employ a pseudo-random number which, with a high probability, resolves the same channel. Additionally, the design of TSCH allows different senders to dispatch information to a specific receiver in the same channel and slot. Then, in order to solve possible collisions within the slot, the standard employs the slotted CSMA-CA algorithm. Another interesting advantage of TSCH protocol is that devices can be members of different *slotframes*. In this case, a device may reserve a reception slot within a *slotframe* of length *n* slots to communicate with a particular group of neighbors and, in turn, the device under consideration reserves a second reception slot within a different *slotframe* of length *p* slots (*n ≠ p*) in order to accomplish data transmission with another group of neighbors. Unfortunately, TSCH presents also several drawbacks. First, it requires extra energy and hardware resources on: (i) senders to appropriately carry out the aforementioned two issues that guarantee the right information transaction with receivers and (ii) senders and receivers in order to perform fast channel switching tasks. Secondly, the standard does not provide any mechanism for the efficient assignment of slots to nodes placed in the same coverage area, and for the initial channel configuration; both features aim at mitigating possible message collisions. In TSCH, this aspect has been considered to be developed by the upper layers. In this context, works in [40] and [41] deal with this concern, presenting a centralized and a distributed algorithm, respectively. In [40], a central node with more capabilities than the remaining network nodes has a complete knowledge of the network topology, which guarantees a quick configuration of the slot and channel employed by each network node. However, the work in [40] penalizes the network overhead and energy consumption due to network reconfiguration tasks whenever a topology change occurs. Concerning the study in [41], it offers a good performance for dynamic scenarios in which there are mobile nodes. However, for static scenarios where nodes remain always at the same location, this solution overloads the network with additional control traffic because devices must periodically transmit information about the slots in use and the channel status. Finally, another significant drawback of TSCH is the inefficient use of the spectrum, which has a negatively impact on robustness and link reliability. The reason is that the IEEE 802.15.4 channels are selected in TSCH without taking into consideration the interference level caused by other wireless technologies as Wi-Fi [42].

### 3.3. WMSN Solutions Developed on Top of the IEEE 802.15.4 PHY and MAC (Beacon Mode) Layers: IEEE 802.15.4e (DSME)

In this category, two scheduling techniques were suggested to solve the collision of beacon frames dispatched by different coordinators (intermediate nodes) or the PAN sharing the same coverage range. The first technique schedules all beacon transmissions within a dedicated period denoted as *Beacon Only Period* (BOP) [43]. In this period, each coordinator device selects a free time-slot which will be employed to transmit its respective beacon frame. Then, once the BOP period is finished, all coordinators (and their associated devices) sharing the same physical medium perform their communications in accordance with the traditional *superframe structure* as defined by the IEEE 802.15.4 standard. However, this approach needs significant modifications in the IEEE 802.15.4 standard to include the BOP period, what restricts its interoperability with the rest of WMSN solutions supported by this same standard. In addition, this approach leads to another important concern. Since simultaneous data transmissions from different coordinators are not scheduled, messages may collide, thus degrading network performance. On the other hand, the second technique, known as *Superframe Duration Scheduling* (SDS), aims at scheduling all the *superframes* dispatched in the same coverage area within a specific interval called *multi-superframe structure*. It consists of a set of consecutive and non-overlapping *superframes*, each one of them from a different coordinator device. So, active periods of each coordinator are sufficiently separated in time, which will, a priori, avoid any data/beacon collision. Note that the *multi-superframe structure* is similar to a TDMA scheduling where each slot contains a *superframe.* The research in [44] shows that in SDS the number of collisions is significantly lower than the one caused by using BOP approaches. This fact notably improves network performance in terms of throughput (number of data messages successfully delivered at the sink). This is one of the reasons why this technique has been the one adopted by the recent standard IEEE 802.15.4e-2012 [18].

Concerning the beacon mechanism, IEEE 802.15.4e-2012 proposes a scheme, called DSME, based on the ideas behind SDS. In DSME, each coordinator periodically transmits a specific beacon frame to inform the nodes in coverage about the exact time instant that the *multi-superframe structure* starts. When a new device with the role of coordinator wants to join the network, it listens to the physical medium waiting for the reception of this beacon frame. Then, once the starting time of the *multi-superframe* is known, the new coordinator searches for an empty slot within the *multi-superframe* to transmit its own beacons and *superframes*. In addition, to further reduce collisions from devices transmitting in the same coverage area, DSME provides two possible solutions, on the one hand, the channel hopping technique described in the previous subsection and, on the other hand, the channel adaptation method based on the concept that nodes always remain in the same channel as long as the interference level experienced is kept within an acceptable range [18,45].

In accordance with DSME, the main problem of this approach arises when network topology changes (e.g., device failures, new nodes willing to join the network or mobility issues) occur in large-scale deployments. Under these conditions and considering that the *multi-superframe structure* is long enough to contain all coordinator's *superframes* in coverage area, the overhead caused by the re-scheduling of the beacon frames and/or the selection of a non-conflicting frequency channel may be quite significant. This fact increases the energy consumption of each device, as well as resource utilization in terms of computing and memory. Another important weakness appears in random topologies when the number of coordinator devices and their location in some sensing areas is a priori unknown by the end-user. In these cases, the time dedicated by the DSME protocol and the energy demanded by the devices may be notably high, especially during the network initialization and configuration phases. On the other hand, a high network density (number of devices per area unit) improves, for instance, routing issues because there are more alternate paths between sources and destinations, which alleviates the impact of the dead intermediate nodes. However, it can negatively affect the delay (time interval since a sender waits for transmitting an information block until this block is finally received by a one-hop neighbor) [46]. Due to the inherent TDMA nature of the *multi-superframe structure*, a coordinator and its associated devices only operate in their corresponding *superframe*, which is in a unique slot belonging to the *multi-superframe*. Then, if the amount of information cannot be dispatched in a unique *superframe*, the set formed by the coordinator and its associated nodes must wait for the next *multi-superframe structure* to continue the transmission of data. Regarding high density networks, *multi-superframe structures* are formed by a greater number of coordinators in coverage, which implies a longer duration of these time structures. Under these conditions, the delay associated to the data transmissions that occupy more than one *superframe* increases as a function of the number of *multi-superframes* employed. Consequently, this fact negatively affects delay. Finally, it should be mentioned that few recent investigations are devoted to studying the network performance using DSME [45,47]. Both works examine diverse metrics of interest such as throughput or energy consumption comparing DSME with the traditional slotted CSMA-CA of the IEEE 802.15.4 beacon-enabled mode under different conditions. However, the topologies used (mainly, the star one) are not consistent with the concept of large-scale WMSNs and, therefore, the results have a questionable validity when such a big network is analyzed.

### 3.4. Overview of Current WMSNs Approaches

Our study reveals that the proposals here discussed exhibit notable limitations and shortcomings that often restrict the number and scope of current and future applications. This fact opens new opportunities that must converge into a unique and more complete solution. In particular, it must fulfill the greatest possible number of requirements for WMSN technology, but considering important concerns as energy consumption and processing/memory capabilities. To this aim, we pay special attention to the IEEE 802.15.5 standard, a non-beacon approach whose promising features offer a great prospect to the WMSNs for its definite penetration in the consumer market. This standard will be explained in detail in the next section. Table 1 summarizes the main features of the different proposals discussed in this work. In this table we also consider the IEEE 802.15.5 standard and the IEEE 802.15.4e specification, which includes a MAC solution aimed at developing WMSNs.

## 4. The IEEE 802.15.5 WPAN Mesh Standard

The IEEE 802.15.5 WPAN mesh standard [2,19,48–50] is aimed at providing traditional WPANs with mesh capability. Even though proposals described in Section 3 can achieve the same performance as IEEE 802.15.5 in some specific aspects (robustness, energy-consumption, scalability, *etc.*), IEEE 802.15.5 satisfies the highest number of WMSN requirements, as shown in Table 1. One of its main benefits is that this standard copes with a wide range of possible applications and this implies, for its better understanding, its division into two different parts denoted as *Low-Rate (LR) WPAN mesh* and *High-Rate (HR) WPAN mesh*. Our attention is focused on the low-rate part which was conceived as a complete mesh support to the IEEE 802.15.4-2006 standard. Thereby, different features such as scalability, robustness, complete autonomy or link reliability were included in the IEEE 802.15.5 recommendation; on-top of the physical (PHY) and MAC (in its non-beacon mode) layers of the IEEE 802.15.4 standard, thus maintaining interoperability. This was possible through the design of new and straightforward primitives that constitute the basis for the initialization, establishment, maintenance and operation of a mesh network. In addition, this standard offers other significant functionalities, such as reliable broadcast and multicast transmission [2], mobility support [51], and a traceroute function, which are useful for those applications that may require them. Nevertheless, the most remarkable functionalities comprised in this recommendation are the synchronous and asynchronous energy saving mechanisms intended to increase network lifetime. As it will be seen along this section, the greater strengths of IEEE 802.15.5 in comparison with the rest of technologies reviewed in this paper are simplicity, low power consumption and bigger improvement of mesh capability, providing an efficient solution for an unlimited number of applications including environmental monitoring, precision agriculture, home building and industrial automation and control, among others.

**Figure 3.**
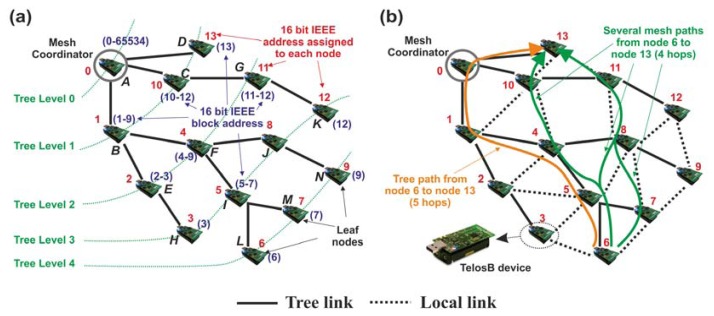
Example of an IEEE 802.15.5 mesh network consisting of 14 TelosB devices: (**a**) Tree formation (function 1) and addressing (function 2); (**b**) Local links generation (function 3) and unicast forwarding (function 4).

As defined by the IEEE 802.15.5 standard, a *mesh* topology consists of a logical tree topology with additional local links among those nodes that are in coverage range (neighbor nodes). The logical tree topology provides transmission of information by means of parent-child pairs, and additional local links allow to select alternative paths between any given source-destination (path redundancy), which improves robustness and link reliability [2]. Under these premises, the appropriate generation and operation of a *LR-WPAN mesh* network is done by means of *four mandatory functions*, namely: (1) start-up and generation of a logical tree multi-hop topology; (2) addressing scheme; (3) generation of local links; and, finally, (4) the unicast data transmission. In particular, functions 1–3 are controlled and coordinated by a unique node called by the standard the *mesh coordinator* (MC), while function 4 must be carried out by every node of the mesh network (including the MC). This means that network nodes (with the exception of end-devices) have the self-organization capability in order to forward the information received from the neighbors, as well as to face different network concerns such as device failures or new devices joining the network, among others. In order to better understand the operation of these four mandatory functions, Figure 3 exemplifies a *LR-WPAN mesh* network composed of 14 TelosB devices [56], where device A is the MC and the rest are nodes that belong to the mesh topology.

Observing Figure 3(a), the creation of the tree network (mandatory function 1) is as follows: the MC is the device in charge of generating the logical tree network (comprising the tree level 0) by firstly setting different configuration parameters such as frequency channel, network identifier (unique for the entire network), addressing scheme (preferably based on 16-bit IEEE addresses), and energy saving mechanism if it was selected by the user of the application. In particular, all nodes are configured with the same single frequency channel selected by the MC, and employ only this specific channel during the entire network operation. However, other wireless technologies such as Wi-Fi may interfere with the mesh network under consideration. In this case, the MC selects a new single frequency channel and reports it to all network nodes. Following with the formation of the logical tree network, new nodes which are located within the MC's coverage range (e.g., nodes B, C, and D) accomplish a discovery process. This is divided into two stages. Firstly, new nodes transmit beacon requests to the MC with the aim of learning about the configuration parameters established by the MC. Secondly, nodes join the network as children nodes of the MC by means of the transmission of association requests, thus fulfilling tree level 1. In turn, these devices become parents (they have the role of *coordinators*, running in non-beacon mode) of new nodes which are out of the MC's coverage range (e.g., nodes labeled as E, F and G –tree level 2–) but can communicate directly with their parents. Consequently, these parents act as routing nodes between the MC and the new children. This process is repeated until all network devices are associated to a parent, forming the different branches of the logical tree multi-hop topology (tree links) whose root is the MC.

Once the network is created, IEEE 802.15.5 executes the second mandatory function, assigning logical addresses to each device. This process is divided into two steps: in the first step, leaf nodes [devices which have no children associated, usually the most remote ones and/or end-devices from the MC, which according to our Figure 3(a) are the nodes labeled as D, H, K, L, M, and N] send their respective parents a message requesting a logical address and additional ones for future use (e.g., new nodes joining the network). In this context, upon joining the network, end-devices dispatch promptly this message. The remaining network nodes trigger a timer to wait for the association of new nodes. Once the timer expires, nodes without associated children become leaf nodes; therefore, they transmit the message asking for the addressing information. When each parent device receives this message from all its children, this device demands its corresponding up-parent the addresses requested by its children and its own address. The process continues for each branch and finalizes when the MC is reached. Then, in the second step, the addresses assignment to every network node starts. The MC is aware of the number of addresses requested by each branch, so this device assigns its children blocks of consecutive 16-bit IEEE logical addresses according to this number. To this aim, the MC sends a control message to each of its children in coverage with the exact number of addresses (block) that they require. After receiving this message, each MC's child extracts its own address from the assigned block (in particular, the first address of the assigned block to identify itself in the network) and, acting as parent, divides the remaining addresses into consecutive sub-blocks which are dispatched to its own children. Again, this process is repeated until the last device(s) of the network (leaf nodes) obtains its address(es).

Figure 3(a) illustrates the second mandatory function. In this Figure, we have supposed for a better comprehension of the reader that every device requests only one address, and no additional addressing space is required in step one. Note that the extra space for addressing allows network nodes to start an individual assignment process with new nodes joining the network. This reduces the overhead required in requesting new addresses directly to the MC and enhances the scalability of the network. Thus, when the second step begins, the MC (which is identified by the 16 bit IEEE logical address 0) assigns each of its branches the corresponding blocks (1–9), (10–12) and (13), respectively. Among all of them, we focus the study on the branch corresponding to node B. So, after receiving block (1–9) from the MC, device B retains the first address for itself (1), and assigns the rest of the block as follows: sub-block (2–3) to node E, and sub-block (4–9) to node F. Following this procedure, the address assignment continues until the devices labeled as H, L, M and N obtain their respective addresses. The key of this function is twofold: (1) every device is identified by a unique address; and (2) the use of 16-bit IEEE logical addresses reduces the number of processing tasks and memory usage, facilitating the management of the routing tables. This entails a clear advantage over other technologies, for instance, 6LoWPAN, which requires header compression and greater memory space to allocate IPv6 addresses in the routing tables; or Zigbee Pro, whose address assignment scheme may cause conflicts due to duplicate addresses.

After the address assignment process, IEEE 802.15.5 activates the generation phase of local links (dotted lines in Figure 3(b)). Note that at this point, the routing tables of every network node only contain information (block address, tree level, hop distance to the device, and relationship between devices: parent, child and neighbor) referred to its up-parent and children (if any) associated to it. Under these circumstances, data transmission can only occur along the tree branches. For this reason, the third mandatory function is aimed at guaranteeing mesh connectivity and link reliability, that is, to enable the communication among devices in coverage range (neighbors) whose relationship is different from parent-child links. To achieve this goal, every node broadcasts a message containing the information stored in its routing table. This message is received by all the nodes in coverage range, which, on the one hand, update their own routing tables and, on the other hand, broadcast a new message with the new updated information. The key design issue of these broadcast transmissions is to fill out the routing tables of all network nodes with information about those placed at a distance of *k* hops (*k* ≥ 2). This approach has two main advantages. Firstly, every device can select any other in its coverage area to transmit data (unicast data transmission –fourth function–, Figure 3(b)), thus sharply increasing the network connectivity in comparison to the plain tree-based topology. To this aim, Figure 3(b) compares tree routing (orange arrow) and mesh routing (green arrow). In this Figure, we can observe that a message transmitted from device 6 (L) to device 13 (D) needs five hops employing tree routing (only one possible path). However, using mesh configuration, it only requires, on average, four hops as well as to enable different alternative paths to connect source-destination. This later feature increases the probability to deliver information even when one or several intermediate nodes die or are overloaded. In this case, the previous node that forwarded data to a dead node, checks its routing table and selects another intermediate node enabling a new path towards the destination. Secondly, unlike traditional reactive routing protocols such as AODV where continuous route discovery processes must be executed, in IEEE 802.15.5 this requirement is eliminated. Instead, nodes dispatch data taking into account the information stored in their routing tables, thus reducing overhead and saving energy. Finally, in order to further improve robustness and link reliability, nodes are able to detect possible link failures. To this end, a node can follow two ways: (i) the node exchanges again local information with its neighbors to update the routing tables or (ii) the node transmits *probe messages* (a very short control message) to test the connectivity with a specific neighbor. In the latter case, when a node detects that the communication with this neighbor fails, then, the node checks it several times by transmitting *probe messages*. If no response is obtained (ack), the node removes the neighbor under consideration from its routing table, and reports the new status to the rest of nodes in coverage range.

In addition to the four mandatory functions described, one of the main contributions of the IEEE 802.15.5 standard is that it provides other significant functionalities, called *non-mandatory* or *enhanced functions*[2]. They are addressed to applications that need specific requirements, such as long-term operation, node mobility, or reliable data transmission, without increasing the implementation complexity in the devices. Among these functionalities, our main interest concentrates on appropriately managing power consumption in the devices so as to save energy, since it is one of the most critical issues in WSNs. In this context, the IEEE 802.15.5 standard provides two solutions denoted as *Synchronous Energy Saving* (SES) and *ASynchronous Energy Saving* (ASES), respectively. SES and ASES are energy saving mechanisms based on a duty-cycle mechanism: periodically, devices that are in a state of full activity (ON), sensing the medium, collecting data and/or forwarding information, switch to a mode of low-power consumption (sleep/OFF) in which communications are temporarily disabled (the radio transceiver is deactivated) and other hardware components, as the micro, are set to the minimum activity. Duty-cycling allows us to extend the device lifetime months or even years, depending on the type of application. This is a clear enhancement in comparison with other technologies or standards, such as Zigbee Pro, which lacks energy-saving mechanisms, or the IP500 proposal, that only facilitates an energy solution to end-devices, obviating this mechanism to the rest of battery-powered devices in the network. This shortcoming forces these nodes to remain in active mode (they never switch to a sleep state), which implies a clear degradation of the device lifetime in comparison with the IEEE 802.15.5 standard. However, duty-cycle mechanisms require the coordination of all the network devices (SES) or at least the nodes involved in the data transmission (ASES), so as to avoid undesirable situations as, for instance, a device sending data to another one in sleeping state. This fact would entail the loss of messages and their later retransmissions, increasing the energy cost and delaying the delivery of messages. This aspect is tackled by SES and ASES under different strategies which are explained in detail in the next subsections.

### 4.1. Synchronous Communications: SES

SES mode [50] is an energy saving mechanism that, thanks to its synchronous character, facilitates the support of delay sensitive applications. In this type of applications, the goal is to guarantee a fast response of the mesh network whenever an event of interest is detected, as well as to ensure a strict delay in the delivery of information at the destination. To this end, in SES mode, all devices are network-wide synchronized, that is, the periods of activity (denoted by the standard as *Active Duration*) and inactivity of each network node (both periods comprise a duty-cycle interval, called *Wakeup Interval*) trigger at the same time. It guarantees that all intermediate devices placed along the source-destination path/-s be in active mode (ON) for the reception and/or forwarding of data. Nevertheless, to obtain strict synchronism during the entire network operation, we should consider two important concerns: (i) the error-prone nature of the wireless channel and (ii) the intrinsic clock drift of devices, that is, a slight random deviation of the clock's oscillator from their nominal frequency, due to impure crystals, temperature changes or variations of power supply and air pressure (e.g., the clock drift value for TelosB devices is, on average, above 30 μs per second [57]). As a result, even if two nodes have the same type of oscillator and are initiated at same time, the difference between both nodes' clocks can become significant as time goes by. Thus, both concerns provoke the desynchronization of the devices' *wakeup intervals* and, as a consequence, failures in the transmission of the information (e.g., loss of data).

**Figure 4.**
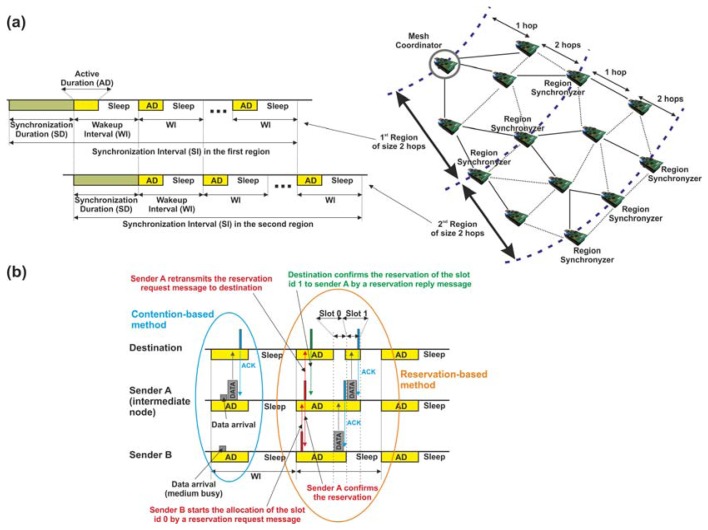
Example of SES (**a**) scheduling and topology; (**b**) transmission methods.

To deal with synchronization, SES is founded on two main aspects: on the one hand, SES divides the mesh network into *n* regions sized in *h* hops (parameter defined by the user) and, on the other hand, SES employs a straightforward synchronization algorithm initiated by the MC. Figure 4(a) exemplifies a network divided into two regions of size *h* equal to 2 hops. The synchronization algorithm operates as follows: the MC begins the synchronization process with every device placed in the first region. This synchronization process is accomplished within a specific period, denoted as *synchronization duration*, where nodes remaining in the active mode dispatch only synchronization messages. In this period, every node synchronizes with its parent device. To this end, parents transmit two consecutive synchronization messages to their children, which, upon receipt, synchronize with the time of its parent device. Children confirm the success of the synchronization process by means of a reply message. Meanwhile, the rest of network nodes out of the first region carry on with their usual operations (sensing, transmission and reception of information or sleep mode) in their corresponding *wakeup intervals.* Then, when the synchronization of the first region finalizes, the same algorithm is executed by the last nodes previously synchronized by the MC. Now, these nodes become synchronizers (namely, *Region Synchronizers*) of the second region, thus finalizing the synchronization process illustrated in Figure 4(a). Note that, regardless of the number of regions contained by the mesh network, each one is synchronized consecutively from the MC's region until the leaf nodes (last region) are reached. An advantage of this synchronization process lies in its reduced network-wide synchronization error which entails that the number of regions can be increased. This represents a clear improvement in the scalability of the network [19]. The synchronization algorithm is run periodically (usually, once each time period denoted as *synchronization interval*, which comprises several *wakeup intervals*) by the MC guaranteeing the synchronization among all network devices and avoiding possible failures in the data transmission due to clock drifts. In addition, this continuous synchronization process helps to solve other issues such as detecting new devices joining the network or nodes leaving the network because of the depletion of their batteries.

SES defines a recovery procedure to cope with the loss of synchronization messages. The recovery is based on the premise of retransmitting the synchronization messages when, for instance, parent nodes do not receive the reply messages from any of their children and the synchronization duration has not expired. Another significant aspect of this procedure lies in its capability of counting the number of synchronization attempts failed at every synchronization interval. In this case, if the number of failed attempts exceeds the threshold value of a parameter defined by the IEEE 802.15.5 standard as *meshMaxLostSynchronization* (default value equal to 3), the recovery method stops the process of synchronization of a node with its parent. Under these circumstances, the node must find another neighbor different from its parent to accomplish the synchronization. To this aim, the node under consideration listens to the physical medium to receive synchronization messages from any neighbor within the synchronization duration. Thereby, once the node is synchronized successfully with one of its neighbors, it will always employ the same neighbor for future synchronization processes.

Regarding data transmission, a key feature of SES mode is its two methods to dispatch information. They differ on how the MAC layer manages the channel access mechanism: while method (1), denoted as *contention-based* method, achieves the transmission of the information by means of a contention-based channel access algorithm, method (2), called *reservation-based* method, operates allocating time slots for exclusive use of nodes. In particular, in method (1), all sender nodes access the physical medium (always within their *active durations*) by using the unslotted CSMA-CA algorithm [20].

Figure 4(b) shows the operation of this algorithm for SES mode. If we study it carefully, two senders in coverage range nodes A and B must transmit a data message to the same destination, which is also in the same coverage area. Firstly, sender A listens to the physical medium. If the medium is free, the message is automatically transmitted. Then, sender B senses the same physical medium which is busy while sender A is dispatching its message. In this case, sender B aborts its transmission attempt and waits for a random time, in order to avoid a collision between messages. Once this time is over and if the *active duration* has not expired yet, sender B triggers the CSMA-CA algorithm again. Otherwise, if the *active duration* has expired, sender B switches to sleep mode and will attempt to transmit the message again during the next *active duration*.

The second mechanism to transmit data, the *reservation-based method*, allows the sources that dispatch critical information (e.g., a node equipped with a physical sensor that detects toxic gases) to establish a dedicated path through several intermediate nodes towards the destination. This dedicated path is accomplished by the thorough coordination of all the nodes that belong to the path with the aim of guaranteeing the transmission of information with strict delay requirements. The contribution of the *reservation-based method* is that it divides the *inactive duration* into slots, each of them dedicated exclusively to transmissions between two nodes in coverage range. Thus, SES takes full advantage of the total bandwidth of each slot, because only one sender and one receiver belonging to the source-destination path dispatch information within their reserved slot without the interference of other transmissions in the vicinity. This is extended along the dedicated path, thus giving support to delay-sensitive applications. Figure 4(b) shows an example of this operation. In this Figure, after the failed transmission attempt by sender B through the *contention-based method*, this node decides to transmit its message to the destination by using the *reservation-based method*. Unlike method (1), we consider that sender B is out of the coverage area of the destination, while node A is in coverage within the destination and sender B, what allows to retransmit messages from sender B to the destination. Under these conditions, sender B initiates the process to allocate dedicated slots within the *inactive duration* by broadcasting a *reservation request* message. The request message contains, among other fields of information, the addresses of the destination and the next intermediate node in the dedicated path (in this example, sender A). In particular, it should be remarked that this request message is subject to the transmission rules of the *active duration* and, as it was mentioned, it employs the unslotted CSMA-CA algorithm to avoid collisions. In conformity with the reservation process, when node A receives the request message, it first checks the destination field included in its header and, since node A is not the destination, this node inspects the message header again to find out the next node. Due to the fact that sender B selected node A as next intermediate node (other neighbor nodes receive the same request, but discard this message), node A continues broadcasting the reservation request towards the destination. In this case, the *reservation request* message from node A has a double function: it forwards the request message to the destination, and, simultaneously, it sends sender B a confirmation of the dedicated link between both nodes. Now, upon the reception of the request by the destination, this node sends node A confirmation of the link by unicasting a *reservation reply* message. At this point, a dedicated path is established between sender B and the destination. So, when the *active duration* expires, sender B starts transmitting its data message within the first slot (slot 0) of the *inactive duration*. Then, once the data has been received by node A, it waits for the following slot (slot 1) to continue dispatching the data message to the destination. The transmission process finishes when node A receives the *acknowledgement* message sent by the destination.

### 4.2. Asynchronous Communications: ASES

**Figure 5.**
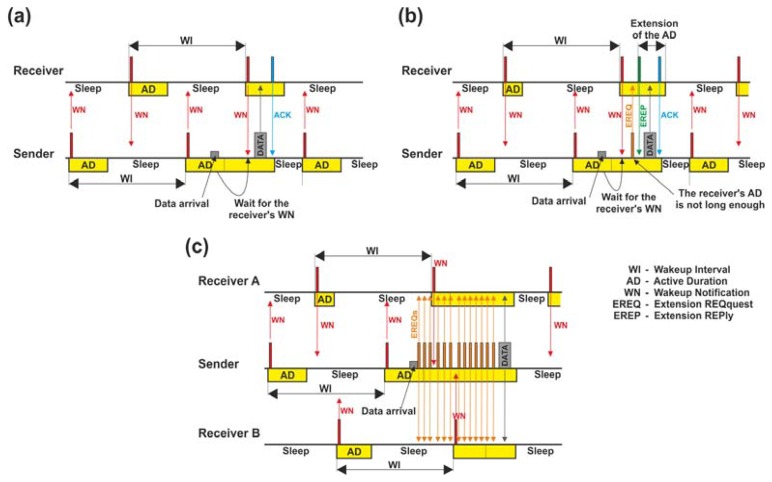
Examples of ASES transmission: (**a**) unicast without extending the receiver's AD; (**b**) unicast by extending the receiver's AD; and (**c**) broadcast.

Unlike SES, ASES is an energy saving mechanism suitable for applications that do not consider strict time requirements, since each node belonging to the source-destination path must contend with its neighbors to transmit data. This results in different delays for each message sent by a same source. In ASES, every network node triggers its own periods of activity and inactivity (ASES uses the same scheme as SES, comprising the *wakeup interval* and *active duration*) regardless of the rest of devices in its coverage range. However, ASES furnishes the functionality of coordination between nodes in order to conduct communications successfully. In particular, ASES must guarantee that a receiver remains in its corresponding AD when a sender transmits a message. To this aim, at the beginning of the *active duration*, every network node individually transmits an announcement message (denoted by the standard as *Wakeup Notification*) so as to inform all the neighbors in its coverage range (and listening to the physical medium) that it *can* receive data. Under this premise, for unicast transmissions, a sender device waits for the reception of the receiver's *wakeup notification* message, in order to later carry out the data transmission by making use of the unslotted CSMA-CA. As Figure 5(a) shows, the sender node extends its own *active duration* waiting for the receiver's WN arrival and, when it occurs, the sender accomplishes the data transmission. Furthermore, ASES provides an additional feature that allows a sender to extend the *active duration* of the receiver by sending an *Extension REQuest* message (EREQ). This happens when the sender detects that the *active duration* of the receiver is not long enough to guarantee the data reception. Again, Figure 5(b) illustrates this functionality: the sender receives the receiver's WN, whose information indicates the sender that the receiver's *active duration* is too short to perform the data transmission. Thus, the sender transmits an EREQ message to the receiver to extend its *active duration*. After the reception of this EREQ message, the receiver acknowledges it by sending a reply message (denoted as *Extension Reply*) and then, the sender node can dispatch data. Finally, broadcast and multicast transmissions can be done in ASES by exploiting the procedure depicted in Figure 5(c). In this Figure, the sender broadcasts several EREQ messages during a time interval longer than one *wakeup interval*. This mechanism must assure that all the receiver nodes (for broadcast transmission, all neighbors in coverage area; for multicast transmission, nodes belonging to the multicast group) are awake when the transmission task is performed (broadcast or multicast). Thus, once the sender stops transmitting EREQ messages, it sends the data message. When the data transmission ends, all nodes (sender and receivers) continue with their normal operation.

## 5. Comparative Analysis based on the Design Guidelines

The valuable benefits of wireless mesh sensor networks have led to the emergence of many standards and proposals promoted not only by the scientific community but also by the commercial and industrial sectors to support real deployments. Among all of them, we agree with the On World study [3] that the most appropriate solution must be a standard validated by the scientific community and commercial sector which satisfies the strict requirements of the low-rate WPAN. In this context and having into account the different WMSN solutions presented in Section 3 together with the IEEE 802.15.5 standard described in Section 4, developers and users should select the approach that best adapts to the necessities of their application. In order to aid the end-user to make the best technological selection, we propose diverse *design guidelines* in accordance with the main WMSN requirements identified in this work (Section 2). These rules are detailed in the next paragraphs and summarized in Table 2.

Firstly, several researchers and task groups, namely 6LoWPAN, ROLL, IP500 Alliance and ISA, have decided on adapting the IPv6 protocol to the IEEE 802.15.4 MAC and PHY layers, mainly to carry out the tasks of addressing and routing. However, the traditional IP protocol stack was designed to be executed on devices such as computers, laptops or tablet-PCs, which have much more hardware and energy resources than sensor nodes. This is the reason why we argue that the operation of WPAN nodes under the IPv6 protocol stack can be inefficient, due to its associated higher implementation complexity and, as a consequence, higher computational and memory costs. On the other hand, one of the major reasons why IPv6 was included in the LR-WPAN field was because it confers the ability to connect nodes directly to external IP-based technologies (e.g., Internet) without considering, a priori, a gateway or proxy device. However, although this facility allows to intercommunicate heterogonous technologies (e.g., Wi-Fi/Ethernet with 6LoWPAN by means of IPv6 addresses) at network layer, the implementation of additional software to adapt the information dispatched by other technologies to WMSNs under the message format defined by IEEE 802.15.4 MAC and PHY layers [8] is not eliminated. This means that part of the gateway functionality must unavoidably be included in each WPAN device, what demands more processing, memory and energy resources.

The next consideration is aimed at selecting the proper addressing scheme for the mesh network (in-network). It is tightly coupled with the routing protocol employed because the type of addressing scheme may imply more complexity of the routing tasks and, as a result, more memory, processing and energy resources in the WPAN devices. In this sense, most of the proposals reviewed in this work have 16-bits logical addresses in their configuration. This value allows us to scale networks up to 65536 devices. Using other addressing schemes (e.g., 64-bit IEEE or IPv6), the maximum number of devices that may form the network sharply increase, so the addressing scheme selected by the user will be closely associated with the scalability of the network to deploy. However, selecting 16-bit IEEE means that routing tables reserve less memory space than in the case of 64-bit or IPv6 addresses and, therefore, the devices are more agile when they handle these tables.

Another important issue is the accessibility and availability of implementations that accelerate the development of new designs and improvements. In this sense, with the exception of ISA SP100.11a and WirelessHART whose documentation (standards) can only be accessed by fee-payment, or IP500 Alliance whose specification has not been published up to date, the remaining approaches presented here are open-standards, which implies that, in principle, any user/researcher has full access to their documentation. On the other hand, Zigbee Pro (through its open-implementation called Open-ZB [58]) and 6LoWPAN/RPL (available for TinyOS [59] and Contiki OS [60]) specifications can be freely downloaded, allowing the users to get their software modules including all their facilities for programming the resource-constrained network devices. In particular, it should be mentioned that Open-ZB solution is fully compliant with the Zigbee Pro specification, although it is not the one provided by Zigbee^®^ Alliance (their implementations are only accessible by fee payment). The remaining proposals do not offer free source-code, excluding the IEEE 802.15.4e and IEEE 802.15.5 standard, which have not had any available implementation yet. On the other hand, new developments and extensions are currently restricted by fee-payment in Zigbee Pro, ISA SP100.11a and WirelessHART standards. This is a clear disadvantage in comparison with the rest of free source-code recommendations, as it may delay the expansion of these fee-payment solutions in the market.

The memory and Central Processing Unit (CPU) utilization is another significant design issue to take into account. WirelessHART and ISA SP100.11a demand a larger amount of computing resources than the remaining solutions here presented in order to implement their entire protocol stack and to achieve the complete functionality of both standards. In the same line, the operation of IEEE 802.15.4e (both TSCH and DSME mechanisms [18]) requires a greater waste in memory and processing capabilities than Zigbee Pro, 6LoWPAN/RPL, IP500, and IEEE 802.15.5 due to the higher complexity of implementation of the TDMA mechanism together with the channel hopping and adaptation techniques. In addition, the operation of the AODV protocol deteriorates the performance of Zigbee Pro through its reactive nature, which forces the nodes to be continuously processing routing information. Furthermore, in the 6LoWPAN/RPL, IP500, and ISA SP100.11a proposals, the implementation of IPv6 connectivity and its associated compression and fragmentation processes entail a higher consumption of hardware resources in the devices comparing with approaches that do not use these functionalities. Finally, IEEE 802.15.5 solves the communications in mesh networks more efficiently, since all mandatory and non-mandatory functions are designed to be implemented in a simple manner (for instance this issue can be demonstrated by means of the synchronization algorithm proposed by SES) into a single layer. This assures, a priori, less utilization of computational and memory resources than the rest of proposals.

The cost and the flexibility of the deployment are two key design decisions which, in many occasions, define the proposal to be employed by the end-user. Both have a strong dependence with other design issues such as the accessibility of the standard or the memory and computing utilization. In this regard, different WMSN manufacturers provide their proprietary hardware/software solutions in accordance with the specifications that they have selected for their implementations. The goal is to penetrate in a specific client-oriented market segment. In particular, specific software and hardware are addressed to non-specialized end-users in WSNs/WMSNs who can easily access this technology, or to experts who can quickly develop their implementations. To enjoy these advantages means an increase in costs because the devices (with its associated software modules) are exclusively provided by the manufacturer. For instance, Dust Networks, a leading company in WSNs for the industrial sector (which was recently acquired by Linear Technology [61]) offers proprietary hardware and software highly compatible with WirelessHART, 6LoWPAN and ISA technologies. However, the cost of its products is up to 30% higher than those provided for example by MEMSIC [62], world-wide manufacturer of wireless sensor hardware (TelosB, MicaZ and Iris platforms) addressed to programming most of the WMSN approaches discussed in this work, or higher than those provided by other small companies and manufacturers [63–65] that market a low-cost and ultra-low energy WSN platforms, offering straightforward solutions for a large number of applications. In addition, the lack of flexibility is another problem associated to these proprietary solutions. This is due to the fact that usually manufacturers offer the user an API at the application level which is insufficient for accessing the full protocol stack, which in turn restricts the possibility of modifying Physical or MAC design parameters, for example. Under these circumstances, WirelessHART and ISA SP100.11a are solutions that follow these concerns, requiring, on the one hand, specific hardware which is capable of supporting all the functionality defined by these standards, and, on the other hand, a fee payment to have free access to the documentation and API implementation. Therefore, the rest of proposals presented in this work are cheaper and more flexible. This implies that their different layers can be programmed and installed in the majority of available wireless sensor platforms of the market.

The next design concern is referred to the number and importance of additional services/functionalities offered by the different proposals which are out of the scope of the requirements exposed in Section 2. In this framework, the IEEE 805.15.5 is the WMSN standard that must allow to design and develop, in our opinion, a larger amount of real-world applications mainly due to its non-mandatory functions (multicast, reliable broadcast or network synchronization, among others). So, for the first time, all these features are integrated in a same recommendation, what makes IEEE 805.15.5 more advantageous against the other proposals that only solve specific concerns of the WMSN communications as, for instance, the message latency.

Another important matter to be addressed by the end-user is the possibility of deploying large-scale networks, which is directly associated to the concept of scalability of the network. In this context, the scalability of 6LoWPAN/RPL, IP500, WirelessHART, ISA SP100.11a and IEEE 802.15.4e is not completely demonstrated due to the lack of studies that assess and validate this issue (for instance, solutions performed over IEEE 802.15.4e only consider, as a maximum value, 25 nodes in their evaluations [45]). However, one of the primary design objectives of the IEEE 802.15.5 standard is related to the scalability, being its performance studied and evaluated in detail in [8]. In particular, authors in [8] compare the IEEE 802.15.5 and Zigbee (AODV) routing schemes in WMSNs composed of topologies ranging from 49 nodes to 784 nodes, revealing a better behavior/network performance for the IEEE 802.15.5. Furthermore, these results confirm the well-known lack of scalability of the AODV protocol.

Finally, as regards power consumption, Zigbee Pro does not currently provide any energy saving mechanism to extend the nodes's lifetime. For WirelessHART and ISA SP100.11a, energy-efficiency is tightly associated to the TDMA scheduling. Nevertheless, the employment of a central node (Network Manager) implies a noticeable increase in the network overhead, which in turn degrades network lifetime, especially for the case of large-scale network deployments. On the other hand, up to the author's knowledge, in the IP500 solution, end-devices remain in sleep state when they do not transmit or receive data, whilst intermediate devices (routers) are always ON, forwarding data or connected to other IP-based technologies. So, intermediate nodes are the “energy bottlenecks” of this proposal. However, this is solved by the usual application field of IP500, focusing only on providing a mesh solution for building automation, in which devices have unlimited access to electric current/power. Concerning 6LoWPAN/RPL, studies in [12,34] propose an asynchronous mechanism whose operation sets that nodes acting as receivers wake up periodically to check the medium for any incoming transmission while senders occupy the entire channel transmitting a *preamble*. However, during the interval when the sender is waiting for the receiver to wake up, senders in ASES mode (IEEE 802.15.5) listen to the physical medium until the reception of the WN's messages. Thereby, ASES allows sender's neighbors to transmit their data within the same period dedicated by the sender node to receive the WN message, thus taking advantage of the available bandwidth. On the contrary, in 6LoWPAN/RPL mechanism, the time period spent by the sender node transmitting its *preamble* cannot be exploited by any other sender's neighbors to transmit data, which involves a significant disadvantage in comparison with ASES. On the other hand, future WMSNs integrating the IEEE 802.15.4e standard in its implementation will increase the network lifetime due to its TDMA-based scheme, in which devices can switch to sleep state after transmitting. Unfortunately, there are not enough studies that evaluate and, therefore, validate the impact of the energy-consumption on each of the approaches based on TSCH and DSME in mesh networks. This restricts the knowledge of its real feasibility on large-scale topologies formed by resource-constrained devices. However, up to date the IEEE 802.15.5 standard offers ASES and SES, which have been evaluated in works [2] and [50], respectively, with promising results for WMSN. In particular, the research in [2] offers a performance evaluation in terms of energy-efficiency of ASES. This study [2] also presents two well-known MAC protocols denoted as Berkeley MAC (BMAC) [66] and XMAC [67], which operate by using their own *low-power listening* techniques, and therefore follow the same premises as the one proposed by 6LoWPAN/RPL. Although results show that the average time nodes remain in active mode is similar regardless of the type of protocol, ASES is the technique assuring a less energy consumption per node, which in turn guarantees a larger network lifetime than BMAC and XMAC protocols.

## 6. IEEE 802.15.5 Discussion and Future Trends

Among all the approaches addressed in this work, we should highlight the IEEE 802.15.5 because it is the standard that appropriately satisfies a greater number of WMSN requirements for low-rate, low-power and resource-constrained devices. This means that the IEEE 802.15.5 considers all those features that turned the IEEE 802.15.4 standard into the most widely adopted WSN solution for one-hop topologies but extending its scope to multi-hop networks and offering mesh capability. Furthermore, since IEEE 802.15.5 includes the IEEE 802.15.4 MAC/PHY layers, it is fully compatible with other proposals that also comprise the IEEE 802.15.4 standard in their protocol stack. As a strong point, IEEE 802.15.5 offers two energy saving mechanisms, ASES, which tackles the traditional sensing WSN applications, and SES, aimed at delaying sensitive applications. This feature, along with its good scalability, assures long-term operation in large-scale mesh networks. Furthermore, this standard also provides additional services (non-mandatory functions), which can be implemented without jeopardizing the processing and memory capabilities of low-cost resource-constrained devices. However, the IEEE 802.15.5 standard presents the following open issues, which reveal future challenges to be explored by the scientific community:

- There are few research works (mostly published in the open literature recently) [2,8,48–51,54,55,68] focused on this standard. Studying them carefully, we can conclude that one of the current weaknesses of IEEE 802.15.5 is the lack of a complete performance evaluation of its main operation and additional functionalities, such as the mobility support or the reliable broadcast transmission.
- To the best of our knowledge, no open-access software implementation and real deployments are available yet to the scientific and professional community. To deal with this negative aspect, software modules should be developed to promote this LR-WPAN mesh solution and to achieve its fast technological transference and penetration in the consumer market.
- Unfortunately, the IEEE 802.15.5 standard is unable to solve some relevant concerns inherited from the broadcast nature of wireless communications. In particular, the main drawback is referred to well-known issues such as the hidden and exposed terminal problems [69]. These problems negatively affect the network performance, causing message losses, increase in latency and energy consumption, poor bandwidth utilization, *etc.* Therefore, more research should be conducted to mitigate or eliminate these problems.
- Even though SES energy saving mechanism is intended for delay-sensitive applications, multimedia services [70] are not supported, a priori, by the standard. This implies considering a new research line, because applications with high traffic demand must be adapted to the WMSN area, paying special attention to significant LR-WPAN concerns as the resource-constrained devices and the available low bandwidth. To this end, SES must be appropriately complemented with new software modules to enable this type of services.
- Finally, aspects such as end-to-end reliability or security are not specified in the current version of the standard [19], what may constitute an interesting topic of research for future work.

## 7. Conclusions

Over the last few years, we have witnessed the success of the IEEE 802.15.4 standard as a recommendation to be employed in LR-WPAN. However, this standard was designed for one-hop data transmission among cost-efficient, ultra-low power and small-size wireless sensor devices. Some recommendations and proposals arose to extend the use of this standard in order to promote multi-hop communications by means of mesh topologies. These proposals provide, among other functionalities, large-scale area monitoring and multiple communication paths between sources and destinations. This type of solutions, denoted as Wireless Mesh Sensor Networks (WMSNs), entails a significant advance in the WSNs arena, enabling a plethora of applications such as protection for fire forest, tele-surveillance in large crops, situational awareness and precision asset location, and health services. This fact involves a strong boost for the penetration of this technology in the consumer market.

In this paper, we reviewed the most competing current mesh proposals to make possible efficient communications in LR-WPAN. The most significant WMSN requirements that allow us to know the benefits and shortcomings of each proposal were also identified. In this sense, as far as the authors know, this is the first study that deals with this concern, which implies a contribution to this technological area. Additionally to this review, we once more inspected the group of proposals following different design guidelines which help to select the best proposal according to the application under consideration. Among all the approaches reviewed, we should highlight the IEEE 802.15.5 standard, a recent WMSN specification that takes full advantage of the IEEE 802.15.4 standard to satisfactorily enforce, among other requirements, interoperability, robustness, link reliability, scalability and, above all, energy-efficiency in mesh topologies formed by WPAN devices. The larger amount of requirements and design guidelines fulfilled by IEEE.802.15.5 implies that the current and future number of applications/services supported by this standard is superior to the majority of approaches presented in this work. Therefore, we also hold a discussion of IEEE 802.15.5, offering hints to improve it with the aim of achieving the first approach that thoroughly guarantees all WMSN requirements and design guidelines. As upcoming research, we plan to continue exploring the WMSN technological field with the goal of, on the one hand, verifying/checking the real penetration in the consumer market of the different standards and recommendations described in this paper, in particular, the IEEE 802.15.5 proposal, and, on the other hand, learning about new WMSN solutions which will emerge in this promising area of knowledge.

## Figures and Tables

**Table 1.** Main features of current wireless mesh sensor network approaches.

**Feature**	**Zigbee^®^Pro [11]**	**6LoWPAN [13]/ RPL [14]**	**IP500^®^solution [15]**	**IEEE802.15.4e™ [18]**	**WirelessHART^®^[16]**	**ISA™ SP100.11a [17]**	**IEEE 802.15.5™ [19]**
**Target Market**	Home, Building and Industrial automation and control; Precision agriculture; Environmental surveillance; *etc.*	Home, Building and Industrial automation and control, smart cities	Home, Building and Industrial automation and control	Home, Building and Industrial automation and control	Industrial automation and control	Industrial automation and control	Home, Building and Industrial automation and control; Precision Agriculture; Environmental surveillance; *etc.*
**802.15.4 standard version**	2003	2003 (RFCs 4919 and 4944) [13]	2006	2011	2006	2006	2006
**Physical Layer**	IEEE 802.15.4 PHY	IEEE 802.15.4 PHY	IEEE 802.15.4 PHY (only 433, 868 and 915 MHz bands)	IEEE 802.15.4 PHY along with channel hopping (TSCH) IEEE 802.15.4 PHY along with channel hopping or channel adaptation (DSME)	IEEE 802.15.4 PHY along with a DSSS/FHSS modulation	IEEE 802.15.4 PHY along with a DSSS/FHSS modulation	IEEE 802.15.4 MAC and PHY
**Power Management**	No (Addressed in future spec.)	No (Addressed in future spec.)	No. Only end devices	Yes	Yes	Yes	Yes
**MAC layer**	Non-beacon mode IEEE 802.15.4 MAC	Non-beacon mode IEEE 802.15.4 MAC	Non-beacon mode IEEE 802.15.4 MAC	Non-beacon mode IEEE 802.15.4 MAC: TSCH Beacon mode IEEE 802.15.4 MAC: DSME	Specific. Based on TDMA	Specific. Based on TDMA and CSMA-CA	Non-beacon mode IEEE 802.15.4 MAC
**Addressing Scheme**	16-bit or 64-bit	128-bit	16-bit for in-network (only WMSN nodes) communication 128-bit for external IP-based communications	16-bit or 64-bit	16-bit or 64-bit	16-bit or 64-bit or 128-bit	16-bit
**Interoperability with other 802.15.4-based technologies**	Yes	Yes	Yes	Yes	No	No	Yes
**Routing Protocol**	Yes. Reactive protocol (AODV)	Yes. Tree-based (RPL)	Unknown (lack of an available standard)	Not Applicable (N/A)	Based on a Network Manager (*central node*)	Based on a Network Manager (*central node*)	Yes. Decisions based on tables formed by mandatory functions 2 and 3 (See Section 3)
**Scalability**	Poor. Due to the AODV protocol	Unknown. Lack of studies	Unknown. Lack of studies	Unknown. Lack of complete evaluation	Good	Good	Good
**Robustness**	Medium Positive: High path redundancy Negative: no support against interferences	Low Negative: No support against interferences and lack of path redundancy due to tree-based routing	Unknown	Medium Positive: Mechanisms to protect against interferences Negative: Poor network adaptation to topology changes	Medium Positive: Mechanisms to protect against interferences Negative: the use of Network Manager, Poor network adaptation to topology changes	Medium Positive: Mechanisms to protect against interferences Negative: Use of Network Manager, Poor network adaptation to topology changes	Medium Positive: Path redundancy Negative: No support against interferences
**Link Reliability**	Medium Positive: Fast path reconfiguration faced to topology changes Negative: Increase in the overhead due to the route discovery process. Trade-off with scalability	Low Positive: Fast path reconfiguration faced to topology changes Negative. Lack of path redundancy and bottlenecks	Unknown	High	Medium. There is path redundancy, although this aspect is low	High	High
**Self-Organization**	Yes	Yes	Yes	Yes	Yes	Yes	Yes
**Support for transport/ application layers**	Yes	Provided by CoRE [33]	Yes	N/A	Yes	Yes	Not considered in the current spec.
**End-to-end Reliability**	No	No	Unknown	N/A	Yes	Yes	Not considered in the current spec.
**Security**	Yes	No	Unknown	N/A	Yes	Yes	No
**Mobility Support**	Yes	Yes	Unknown	No	Yes	Yes	Yes [2,51]
**Available Evaluation**	Yes [28,30,31]	Yes [34–36,52]	No	Yes. TSCH: [40,41] DSME: [45,47]	Yes [23,24,27,53]	Yes [26–28]	Yes [2,8,49,50,54,55]

**Table 2.** Decision issues and classification of the current WMSN approaches.

**Decisions**	**Zigbee^®^Pro [11]**	**6LoWPAN [13]/ RPL [14]**	**IP500^®^solution [15]**	**IEEE802.15.4e™ [18]**	**WirelessHART^®^ [16]**	**ISA™SP100.11a [17]**	**IEEE 802.15.5™ [19]**
**Use of IPv6**	No	Yes	Yes	No	No	Yes	No
**In-network addressing scheme**	16-bit	128-bit (IPv6)	16-bit	16-bit	16-bit	16-bit	16-bit
**Available Source Code**	Open-ZB [58]	TinyOS's 6LoWPAN/ RPL [59] ContikiRPL [60]	No	Open issue	No	No	Open issue
**Fee-payment contributions**	Yes	No	Yes	No	Yes	Yes	No
**Memory and CPU usage**	Medium	Medium	Medium	High	High	High	Low
**Cost**	Medium-Low	Low	Medium	Low	High	High	Low
**Flexibility**	High	High	High	High	Low	Low	High
**Additional services**	Multicast transmission Reliable broadcast transmission	IPv6-based connectivity Multicast transmission	IPv6-based connectivity	Delay-sensitive application support Deterministic latency	Delay-sensitive application support Deterministic latency Multicast transmission Reliable broadcast transmission	IPv6-based connectivity Delay-sensitive application support Deterministic latency Multicast transmission Reliable broadcast transmission	Reliable broadcast transmission Trace route function Multicast transmission Delay-sensitive application support Overall Network Synchronization and deterministic latency (SES mode)
**Large-Scale Networks**	No	Unknown	Unknown	Unknown	Yes	Yes	Yes
**Energy Saving support for battery-powered devices**	No	No (asynchronous solution based on low-power listening)	No. Only end-devices and based on a duty-cycle mechanism	Yes (TSCH and DSME)	Yes (In TDMA scheme, nodes transmit in their corresponding slot; the remaining slots, nodes in sleep state)	Yes (In TDMA scheme, nodes transmit in their corresponding slot; the remaining slots, nodes in sleep state)	Yes (SES and ASES)
